# MiR-21 promotes intrahepatic cholangiocarcinoma proliferation and growth *in vitro* and *in vivo* by targeting PTPN14 and PTEN

**DOI:** 10.18632/oncotarget.3465

**Published:** 2015-02-28

**Authors:** Li-Juan Wang, Chen-Chen He, Xin Sui, Meng-Jiao Cai, Cong-Ya Zhou, Jin-Lu Ma, Lei Wu, Hao Wang, Su-Xia Han, Qing Zhu

**Affiliations:** ^1^ Department of Oncology, the First Affiliated Hospital of Medical school of Xi'an Jiaotong University, Xi'an, Shaanxi Province, P.R. China; ^2^ Center of Radiotherapy, Shaanxi Provincial Tumor Hospital, Shaanxi Province, P.R. China

**Keywords:** Intrahepatic cholangiocarcinoma, miR-21, PTPN14, PTEN, tumorigenesis

## Abstract

Intrahepatic cholangiocarcinoma (ICC) constitutes the second-most common primary hepatic malignancy. MicroRNAs (miRNAs) play important roles in the pathogenesis of ICC. However, the clinical significance of miR-21 levels in ICC remains unclear. Here, we investigated the role of miR-21 in ICC and found that its expression was significantly upregulated in serum of ICC patients. Serum miR-21 levels robustly distinguished ICC patients from control subjects. Further experiments showed that inhibition of miR-21 suppressed ICC cell proliferation *in vitro* and tumor growth *in vivo*. Specifically, inhibition of miR-21 induced cell cycle arrest and apoptosis. Moreover, *PTPN14* and *PTEN* were identified as direct and functional targets of miR-21. Finally, we showed high expression levels of miR-21 were closely related to adverse clinical features, diminished survival, and poor prognosis in ICC patients. This study revealed functional and mechanistic links between miR-21 and tumor suppressor genes, *PTPN14* and *PTEN*, in the pathogenesis of ICC. MiR-21 not only plays important roles in the regulation of cell proliferation and tumor growth in ICC, but is also a diagnostic and prognostic marker, and a potential therapeutic target for ICC.

## INTRODUCTION

Intrahepatic cholangiocarcinoma (ICC) is relatively rare in most populations, but constitutes the second-most common primary hepatic malignancy [1-3], with increased worldwide incidence over the past three decades [4, 5]. Although the incidence of ICC in the United States and Europe is still relatively low, ICC rates are extremely high in China, where liver fluke infection is endemic [6-8]. Adjuvant therapy is often not efficacious for ICC: the only potential curative treatment being surgical resection. Unfortunately, the great majority of ICC patients are diagnosed at late clinical stages, when curative surgery is not a viable option [9, 10]. Survival times for these patients is typically measured in months [4, 11-13]. Moreover, diagnostic techniques are plagued by low specificity in early stage ICC [4]. Given the increased incidence of ICC, its manifest aggressiveness and poor outcomes, further studies clarifying diagnostic and prognostic factors are warranted.

MicroRNAs (miRNA) comprise a group of small non-coding RNAs (approximately 22 nucleotides in length, [14]), transcribed from non-protein coding genes or introns, and which regulate gene expression through repressing translation or inducing mRNA degradation by binding to complementary sites in 3′-untranslated regions (3′-UTRs) [15]. MiRNAs regulate the expression of a wide variety of target genes, and aberrant expression of miRNAs functions as tumor suppressors or oncogenes according to the role of their target genes [16, 17]. Increasingly, miRNAs have been observed in various types of cancer and are involved in modulating cancer cell behavior, including cell proliferation and apoptosis, cell cycle, cellular migration, and cell invasion [18-20]. More importantly, some studies have identified that tumor-derived miRNA can be detected in blood and appear to be stably protected from circulatory endogenous ribonuclease activity [21]. Several aberrantly-expressed serum and tissue miRNAs have been employed as diagnostic or prognostic indicators in multiple cancer types [22-24]. These data are consistent with the hypothesis that miRNAs play important roles in the development and progression of human cancers.

Recently, the role of miRNAs in the establishment and progression of ICC has become evident, and some miRNAs have been identified as tumor suppressor genes or oncogenes in ICC [25, 26]. Specifically, miR-21 is frequently implicated, being upregulated during tumor progression and also associated with poor survival in very diverse types of malignancy. Moreover, miR-21 may play a crucial role in modulating the expression of gene products involved in phenotypic characteristics of cancer cells such as cell proliferation, cell apoptosis, and cell cycle [27, 28]. MiR-21is overexpressed in ICC cell lines [29], and many tumor suppressor mRNAs have been identified as direct targets of this mRNA, including programmed cell death 4 (*PDCD4*) [30], phosphatase and tension homolog (*PTEN*) on chromosome 10 [31], tissue inhibitor of metalloproteinase 3 (*TIMP3*) [30], and reversion-inducing cysteine-rich protein with kazal motifs (*RECK*) [32]. However, the clinical significance of circulating miR-21 levels in ICC remains unclear and biological functions of miR-21 and its targets in ICC have not been characterized.

In this study, we report that serum miR-21 levels can be utilized as a diagnostic marker in ICC. Furthermore, for the first time, we describe the effects of miR-21 on its potential target genes protein tyrosine phosphatase non-receptor type 14 (*PTPN14*), and *PTEN* in various malignant phenotypes of ICC cells in *vitro* and in *vivo*. Finally, we detected the expression of miR-21, *PTPN14* and *PTEN* in tissues of ICC patients, and characterized the clinicopathological correlation of miR-21, *PTPN14*, and *PTEN* in ICC. To the best of our knowledge, the present work is the most comprehensive and systematic investigation of the clinicopathological correlations and biological functions of miR-21 and its direct targets *PTPN14* and *PTEN* in the tumorigenesis and progression of ICC.

## RESULTS

### MiR-21 expression in ICC cell culture medium

**Figure 1:**
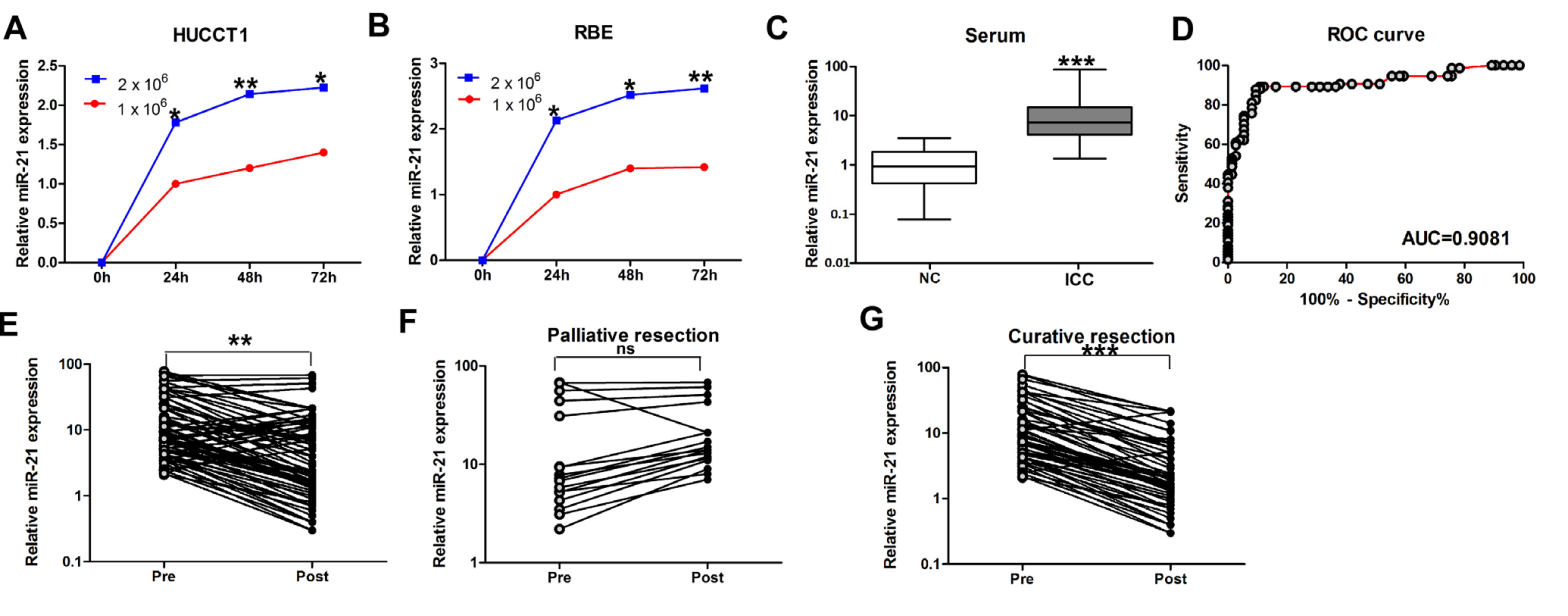
Expression of miR-21 in culture media of ICC cell lines and serum samples (A) and (B) MiR-21 levels in the media of both HUCCT1 and RBE increased with increased cell counts and longer incubation intervals. (C) Serum levels of miR-21 in normal controls and ICC patients. Boxes represent interquartile range, and the horizontal line across each box indicates median value. (D) Serum miR-21 yielded an area under the curve (AUC) value of 0.9081, with 87.8% sensitivity and 90.5% specificity in distinguishing ICC patients from normal control subjects. (E) Comparison of serum miR-21 levels from all ICC patients (n=74). (F) Comparison of serum miR-21 in 17 ICC patients who underwent palliative resection. (G) Comparison of serum miR-21 levels in 57 ICC patients who underwent potentially curative surgeries.

MiR-21 has been identified as a secreted miRNA in multiple cancers types [33, 34]. We investigated whether miR-21 also acted similarly in ICC and was secreted into culture medium by HUCCT1 and RBE ICC cell lines. As anticipated, miR-21 was detected in the culture medium from each cell line and increased over time (*P* < 0.05; Figure 1A, HUCCT1; Figure 1B, RBE). MiR-21 levels also increased with elevated numbers of tumor cells (*P* < 0.05; Figure 1A, HUCCT1; Figure 1B, RBE). These results suggest miR-21 is a secretory miRNA in ICC cell lines.

### Serum miR-21 expression in negative controls and patients with ICC

We next quantified circulating miR-21 levels in serum samples from ICC patients (n = 74) and healthy control subjects (n = 74). We found that miR-21 levels were statistically significantly elevated in the sera of ICC patients (*P* < 0.001; Figure 1C). Based on these results, we focused our study on the efficacy of serum miR-21 as a diagnostic and prognostic biomarker in patients with ICC in the following experiments.

We generated ROC curves to assess the potential usefulness of serum miR-21 as a noninvasive biomarker for early diagnosis of ICC. Our ROC analyses revealed that serum miR-21 levels were robust in discriminating patients with ICC from healthy control subjects with an AUC value of 0.9081 (Figure 1D). Using a cutoff value of 2.971, the sensitivity, specificity, and positive and negative predictive values were 87.8, 90.5, 90.2 and 88.2%, respectively, to identify a patient with ICC.

We then analyzed paired pre- and postoperative serum samples in the subset of 74 ICC patients who underwent surgical resection of their tumor. In the 74 ICC patients, 57 underwent potentially curative resection, whereas 17 had multiple hepatic metastases and underwent palliative resection. We found that serum levels of miR-21 were statistically significantly diminished after surgery in the same subset of patients (*P* < 0.01; Figure 1E). However, when the data were analyzed based on potentially curative or palliative surgical groups, postoperative reductions in serum miR-21 levels occurred in the group of patients who received potentially curative surgeries (*P* < 0.001; Figure 1G). In contrast, no statistically significant difference was observed in miR-21 levels before or after surgery in the group of patients with palliative resections (Figure 1F). Taken together, these data underscore the importance of serum miR-21 expression as a highly specific biomarker for the diagnosis of ICC.

### Effect of miR-21 inhibition on multiple malignant phenotypes of ICC cells

**Figure 2:**
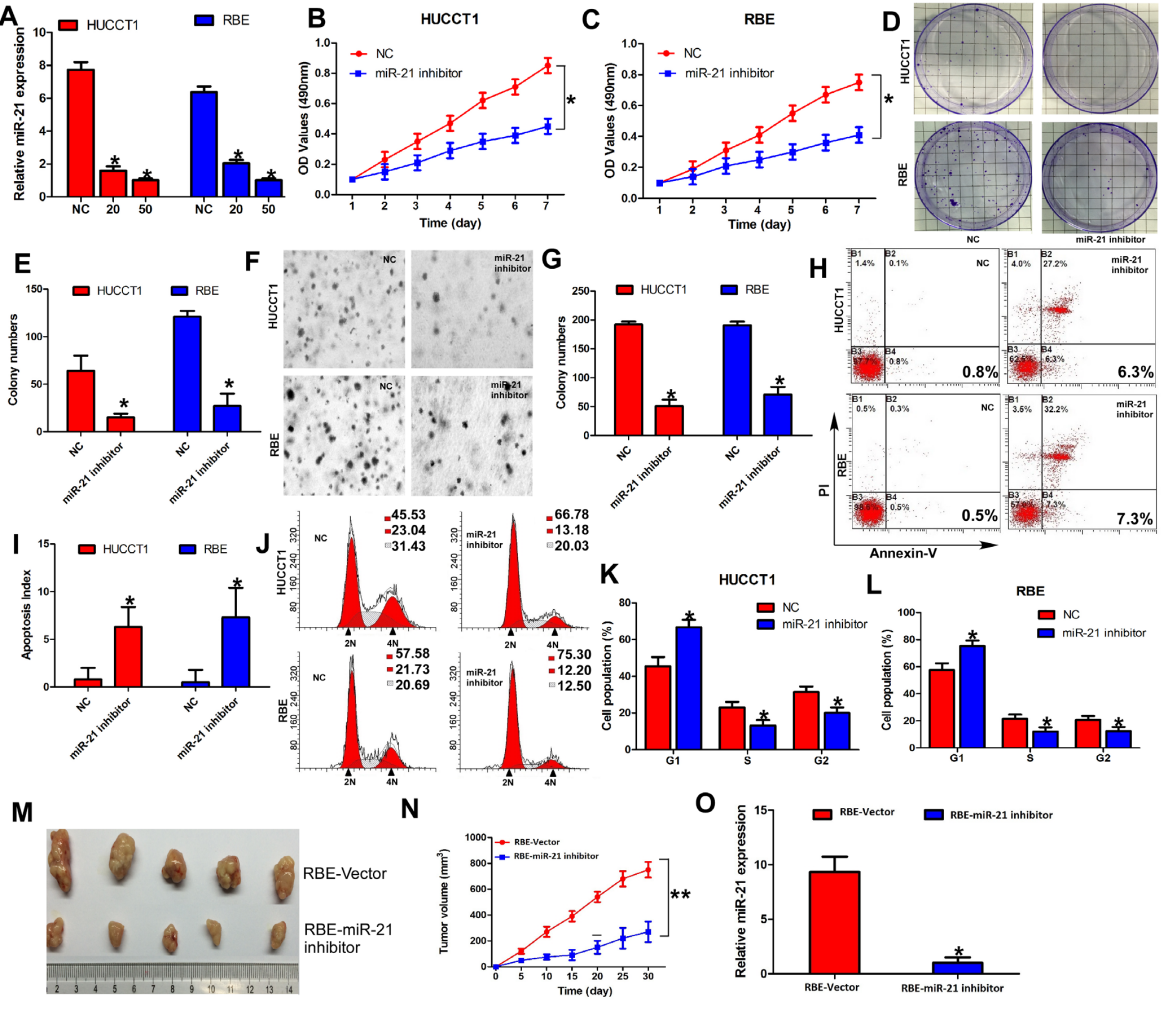
Inhibition of miR-21 inhibits malignant phenotypes of ICC cells *in vivo* and *in vitro* (A) Expression of miR-21 in HUCCT1 and RBE with increasing amounts of miR-21 inhibitor (20 and 50 nM) was validated by RT-qPCR. (B) and (C) Cell growth analyzed by MTT assays. (D) and (E) Representative results of colony formation. (F) and (G) Representative results of soft agar colony formation. The number of colonies counted was that of an entire well and the error bars represent mean ± SD from three independent experiments. (H) and (I) Effects of miR-21 on cell apoptosis of HUCCT1 and RBE cells. (J), (K), and (L) Effects of miR-21 on cell cycle of HUCCT1 and RBE cells. (M) and (N) RBE-Vector and RBE-miR-21 inhibited cells were injected into the hind limbs of nude mice (n = 5). Tumor volumes were measured on the indicated days. Data points are presented as the mean tumor volume ± SD. (O) RT-qPCR analysis of miR-21 expression in tissues of resected tumor formed from RBE-Vector and RBE-miR-21 inhibitor.

Given that a single miRNA type can negatively regulate hundreds of target genes simultaneously, we speculated that miR-21, an important oncogenic miRNA, might affect diverse malignant behaviors of ICC cells. In order to evaluate the multiple effects of miR-21 on malignant phenotypes in ICC cells, we silenced miR-21 expression in HUCCT1 and RBE cells by transfecting has-miR-21 inhibitor oligonucleotides. Transfection efficiency was confirmed through real-time PCR (both *P* < 0.05; Figure 2A). MTT assays revealed that miR-21 inhibitor-transfected HUCCT1 and RBE cells exhibited significantly decreased growth rate than normal control (NC)-transfected cells (*P* < 0.05; Figure 2B and Figure 2C). Colony formation assays also showed that silencing miR-21 expression resulted in significant tumor growth inhibition (*P* < 0.05; Figure 2D and Figure 2E). We next examined the effect of miR-21 on anchorage-independent growth ability of ICC cells by using the soft agar assay. Inhibiting miR-21 expression significantly reduced growth of HUCCT1 and RBE cells (*P* < 0.05; Figure 2F and Figure 2G).

To explore possible mechanisms of miR-21 function in controlling ICC cell proliferation, we measured the effect of miR-21 on apoptosis of ICC cells. Results showed that the rate of early apoptosis was significantly higher when miR-21 was inhibited in HUCCT1 and RBE cells (*P* < 0.05; Figure 2H and Figure 2I). Then, we determined the distribution of cells within cell cycle stages by flow cytometry. Cells treated with miR-21 inhibitors displayed a significant increase in the percentage of cells in the G1/G0 peak and a decrease in the percentage of cells in the S and G2/M peak (Both *P* < 0.05; Figure 2J, Figure 2K and Figure 2L). These results suggested that miR-21 could promote cell proliferation by regulating the cell cycle and apoptosis.

To confirm miR-21 function in *vivo*, we engineered RBE cells to stably downregulate miR-21 expression and performed a tumorigenesis assay in nude mice. The cells were injected into the flanks of nude mice. Tumor sizes were measured every 5 days; after 30 days, the mice were sacrificed and tumors were collected. Analysis of tumor growth curves confirmed that the tumors in the RBE-miR-21 inhibitor group grew significantly more slowly than those in RBE-Vector group (P < 0.05; Figure 2M and Figure 2N). The expression of miR-21 in the tumors was measured by RT-qPCR, which indicated that miR-21expression was significantly lower in the RBE-miR-21 inhibitor group than the RBE-Vector group (*P*< 0.05; Figure 2O). Taken together, these findings identify that miR-21 plays crucial roles in multiple malignant phenotypes of ICC *in vivo* and *in vitro*.

### *PTPN14* and *PTEN* are direct targets of miR-21 in ICC cells

**Figure 3:**
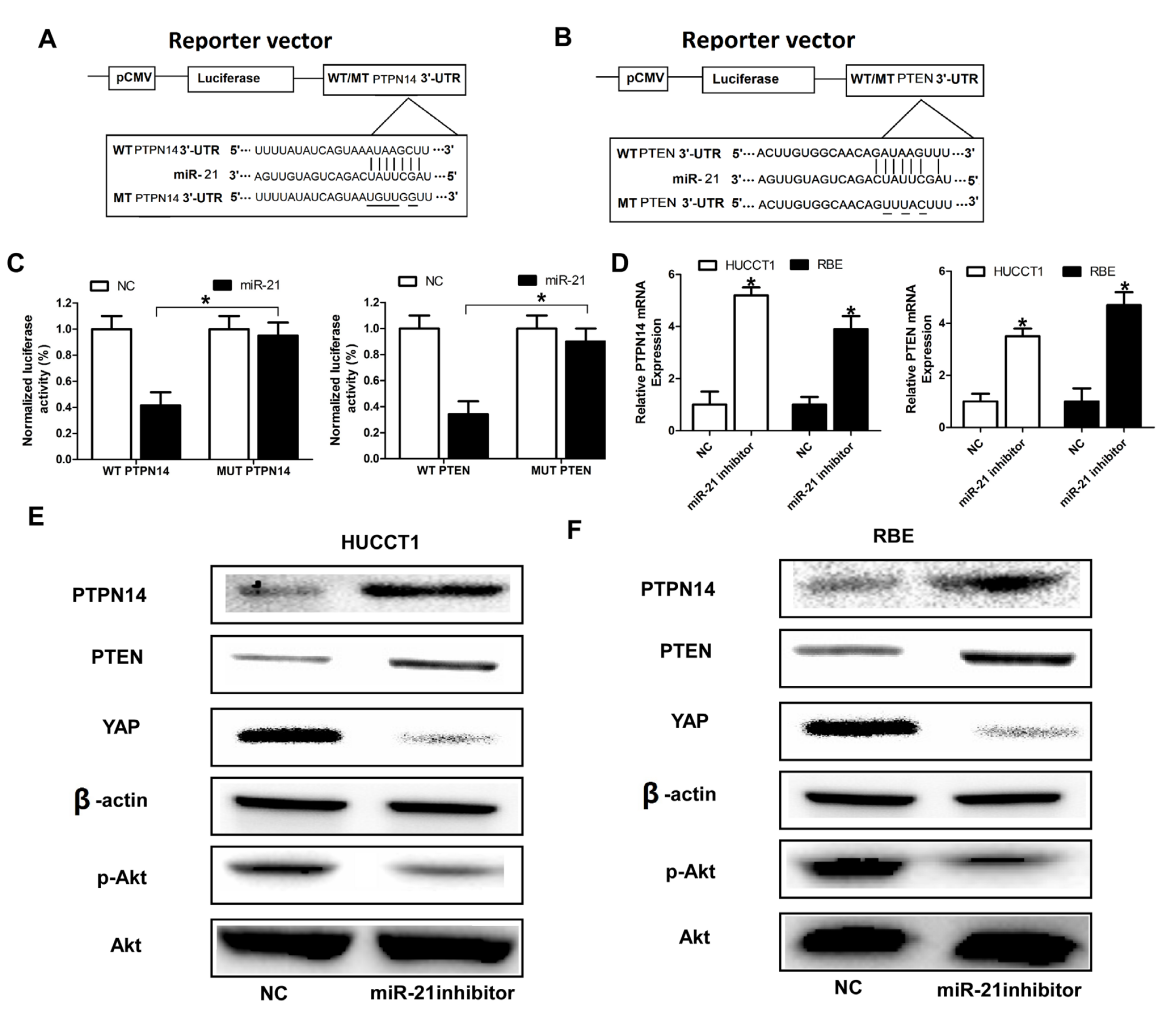
*PTPN14* and *PTEN* are direct targets of miR-21 (A) and (B) MiR-21 and its putative binding sequence in the 3′-UTR of *PTPN14* and *PTEN*; diagrammatic representation of the luciferase reporter plasmids with WT and MT *PTPN14* and *PTEN* 3′-UTR. (C) Relative luciferase activity in 293T cells after transfection with WT or MT *PTPN14* or *PTEN* 3′-UTR plasmids co-transfected with miR-21 inhibitors. (D), (E), and (F) MiR-21 inhibitors promoted the expression level of PTPN14 and PTEN at the mRNA level and protein level in HUCCT1 and RBE cells. Three independent experiments were performed in duplicate. Data are presented as mean ± SD. Two-tailed Student's t test was used. * *P*< 0.05.

To identify the potential targets of miR-21 that might contribute the malignant phenotypes of ICC cells, we performed an unbiased computational screen by integrating the results of multiple prediction algorithms (Targetscan, Pic Tar and miRanda). We identified *PTEN* and *PTPN14* as theoretical target genes of miR-21. Luciferase reporter assays demonstrated that miR-21 significantly repressed activity of reporter vectors harboring wild-type 3′-UTRs of *PTPN14* and *PTEN*, whereas mutations of putative miR-21-bingding sites in these 3′-UTR regions abrogated the inhibitory effects of miR-21 (Figure 3A, Figure 3B, Figure 3C). Furthermore, RT-qPCR and Western blot analyses showed that mRNA and protein levels of PTPN14 and PTEN were dramatically upregulated in HUCCT1 and RBE cells when miR-21 expression was inhibited (Figure 3D, Figure 3E, Figure 3F). We also investigated the effect of miR-21 on Yes-associated protein 1 (YAP) which has been demonstrated to be directly regulated by PTPN14 [35, 36] and Akt which was the downstream of PTEN. As anticipated, the expression of YAP, and pAkt was significantly downregulated in HUCCT1 and RBE cells when miR-21 expression was inhibited (Figure 3E and Figure 3F).

### Repression of PTPN14 and PTEN signaling plays crucial roles in miR-21-induced malignant phenotypes of ICC cells

**Figure 4:**
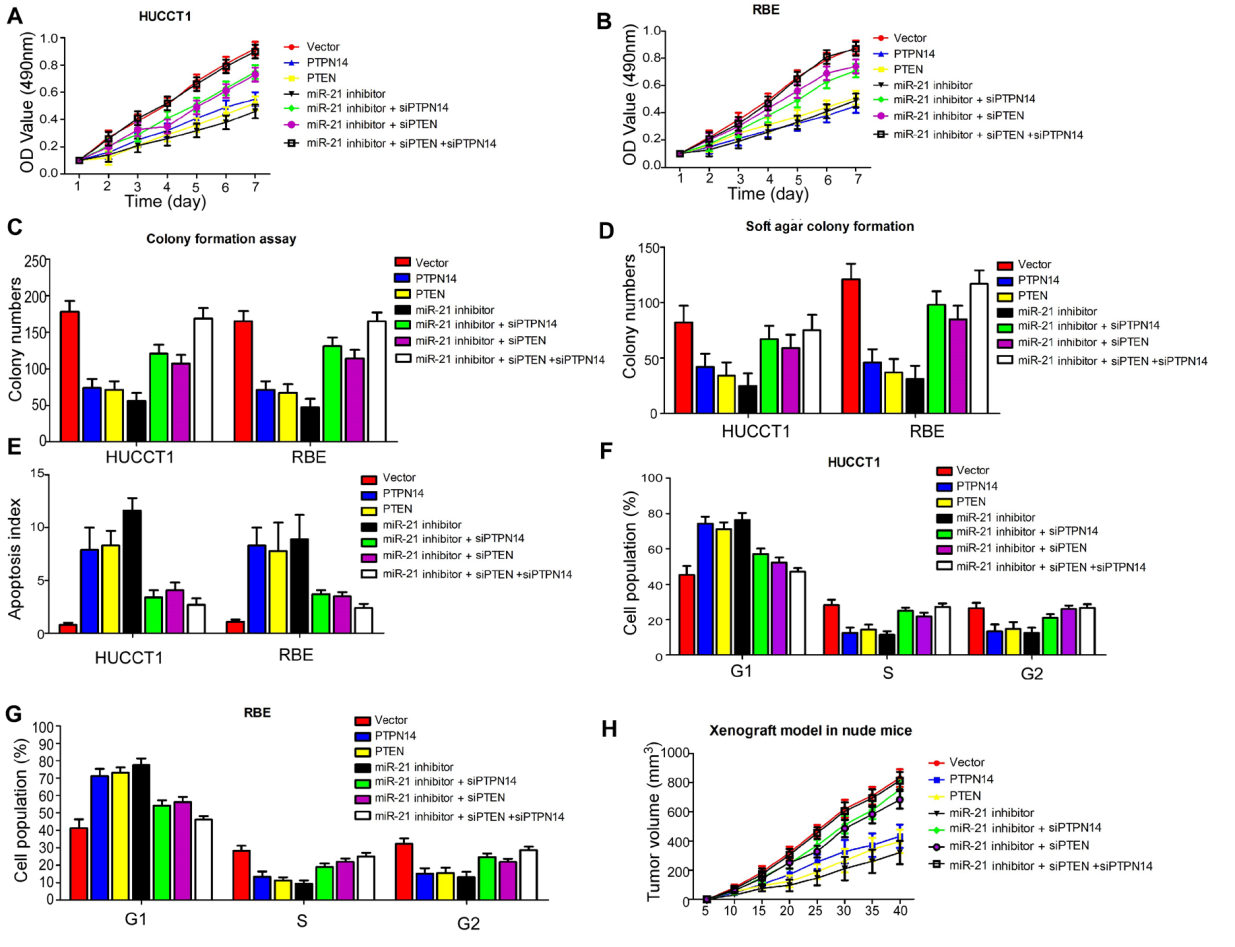
Both gain- and loss-of-function studies showed that both PTPN14 and PTEN abrogate the suppressive roles of miR-21 in ICC cell proliferation and cell growth *in vivo* and *in vitro* (A), (B) Cell proliferation; (C) Colony formation assay; (D) Soft agar colony formation assay; (E) Cell apoptosis assay; (F), (G) Cell cycle assay; and (H) Tumorigenesis assay indicate silencing *PTPN14* and/or *PTEN* could rescue the inhibitory effects of miR-21-suppressors on HUCCT1 and RBE cells.

Next, we asked whether these newly identified target genes of miR-21 were responsible for the aggressive phenotypes of ICC cells. To explore the role of *PTEN* and *PTPN14* in ICC cells, we constructed plasmid vectors expressing *PTPN14 and PTEN* without the 3′-UTR, and confirmed their efficacy on levels of PTPN14 and PTEN proteins in REB cells (Supplementary Figure 2). We then examined whether overexpressed PTPN14 and PTEN would mimic the effects of inhibition of miR-21 in HUCCT1 and RBE cells. As expected, compared to the control group, ectopic delivery of PTPN14 and PTEN displayed a similar effect on cell proliferation in HUCCT1 and RBE cells (Figure 4A and Figure 4B). In addition, silencing the single-gene expression of *PTPN14* or *PTEN* in miR-21 inhibitor-transduced cells attenuated the inhibitory effect on cell proliferation of HUCCT1 and RBE cells. More importantly, we found that when the expression of *PTPN14* and *PTEN* were silenced at the same time, inhibition of cell proliferation induced by miR-21 inhibitors was remarkably attenuated (Figure 4A and Figure 4B). Similarly, we found silencing *PTPN14* and/or *PTEN* could rescue the effects of miR-21 inhibitors on HUCCT1 and RBE cells, as revealed by colony formation assays (Figure 4C), soft agar colony formation assays (Figure 4D), cell cycle assays (Figure 4F, Figure 4G), and tumorigenesis assays (Figure 4H). However, we also found silencing *PTPN14* and/or *PTEN* could inhibit apoptosis rates of HUCCT1 and RBE cells induced by miR-21 inhibitors (Figure 4E). These results indicate that *PTPN14* and *PTEN* may serve as downstream functional targets of miR-21.

### Expression of miR-21, PTPN14 and PTEN in ICC tissues

**Figure 5:**
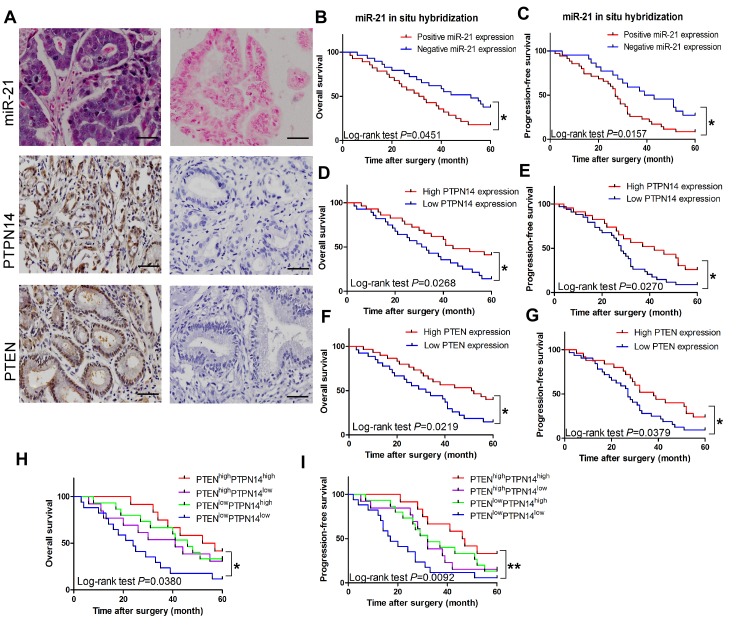
Clinical significance of miR-21, *PTPN14* and *PTEN* in ICC (A) *In situ* hybridization for miR-21 and IHC for PTPN14 and PTEN in the tissues of ICC patients. Positive representation of miR-21, PTPN14, and PTEN are on the left half of Figure 5A, negative presentation of miR-21, PTPN14, and PTEN are on the right half of Figure 5A. Magnification: x 200; Scale bar: 50 μm. (B) and (C) Kaplan-Meier plots representing probabilities of progression-free and overall survival in ICC patients according to expression level of miR-21. (D) and (E) Kaplan-Meier plots representing probabilities of progression-free and overall survival in ICC patients according to expression level of *PTPN14*. (F) and (G) Kaplan-Meier plots representing probabilities of progression-free and overall survival in ICC patients according to expression level of *PTEN*. (H) and (I) Kaplan-Meier plots representing the probabilities of progression-free and overall survival in ICC patients according to expression level of *PTPN14* and *PTEN*.

To determine the prevalence and clinical significance of miR-21 and its direct targets *PTPN14* and *PTEN* in ICC, we assessed the expression of miR-21, *PTPN14* and *PTEN* by miRNA *in situ* hybridization and IHC staining in tumor tissue samples from 57 ICC patients who received curative resection. Among the 57 patients, 28 were positive for miR-21 expression (29 negative); 28 were positive for either nuclear or cytoplasmic PTPN14 expression (29 negative); and 25 were positive for PTEN expression (32 negative) (Figure 5A).

The relationships between miR-21 expression and clinicopathologic factors were analyzed using the Chi-Square test and results are shown in Table 1. Our results showed that the expression of miR-21 was significantly associated with clinical stage at diagnosis and tumor differentiation status (*P* = 0.002 and *P* = 0.045, respectively), but was not significantly correlated to gender, age, or tumor origination (Table 1).

**Table 1:** Relationship of tissue miR-21 expression and clinicopathologic parameters

Variables	No.	miR-21 positive	miR-21 negative	*p*
**All patients**	57	28	29	
**Age at diagnosis(years)**				0.274
**≤60**	21	8	13	
**>60**	36	20	16	
**Gender**				0.596
**Male**	32	17	15	
**Female**	25	11	14	
**Clinical stage at diagnosis**				
**I**	5	1	4	0.002*
**II**	22	5	17	
**III**	25	18	7	
**IV**	5	4	1	
**Differentiation**				
**Well**	18	5	13	0.045*
**Moderate/poor**	39	23	16	
**Tumor origination**				
**Left**	21	8	13	0.317
**Right**	32	17	15	
**Bilateral**	4	3	1	

* Significant relation of clinical factors with overall survival

### Association of tissue miR-21, *PTPN14* and *PTEN* expression with prognosis in patients with ICC

We performed Kaplan-Meier analyses to determine whether tissue miR-21 expression was associated with overall and progression-free survival in ICC patients. Patients with high tissue miR-21 expression exhibited poor 5-year overall and progression-free survival rates in comparison to patients with low miR-21 expression. The differences were statistically significant (*P* = 0.0451 and *P*= 0.0157, respectively; Figure 5B and Figure 5C). Conversely, patients with elevated *PTPN14* or *PTEN* expression displayed improved 5-year survival rates compared to patients with diminished *PTPN14* or *PTEN* expression (both *P* < 0.05; Figures 5D and Figure 5G). In accord with the expression status of PTPN14 and PTEN, these ICC patients were divided into four groups: (1) PTEN^high^/PTPN14^high^ (n=12); (2) PTEN^high^/PTPN14^low^ (n=13); (3) PTEN^low^/PTPN14^high^ (n=15); (4) PTEN^low^/PTPN14^low^ (n = 17). Kaplan-Meier analysis revealed that those patients bearing PTEN^high^/PTPN14^high^ tumors had significantly longer overall survival (*P* = 0.0380) and progression-free survival (*P* = 0.0092) than patients whose tumors overexpressed either one or neither of the proteins (Figure 5H and Figure 5I). More importantly, we found miR-21 inversely correlated with PTPN14 and PTEN in the tissues of ICC patients (Supplementary Figure 1). These results indicate that ICC tumors overexpressing both PTEN and PTPN14 are less aggressive. As previously indicated, both *PTEN* and *PTPN14* were direct targets of miR-21 and our results proposed a prognostic role of miR-21 in ICC. Thus, we further investigated the prognostic value of quantification of miR-21expression in ICC.

To test the prognostic value of high tissue miR-21 expression, a Cox proportional hazard model was used. Univariate analysis identified clinical stage at diagnosis, differentiation status and miR-21 expression as poor prognosticators for overall survival and progression-free survival (all *P* < 0.05), whereas age, gender and tumor origination were not significantly associated with overall survival and progression-free survival (Tables 2 and 3). Patients with high tissue miR-21 expression tended to have a higher risk of ICC progression and death compared to patients with low tissue miR-21 expression, with unadjusted HR being 4.736 (*P* = 0.016) and 3.271 (*P* < 0.001), respectively. To test whether prognostic value of high tissuemiR-21 expression was independent of other risk factors for poor overall and progression-free survival, a multivariate analysis was performed using a Cox proportional hazard model. Multivariate analyses including age, gender, tumor origination, clinical stage at diagnosis, differentiation status, and miR-21 expression demonstrated that high tissue miR-21expression was an independent predictor for poor overall- and progression-free survival in ICC patients (HR = 3.519 CI = 1.411–5.702, *P* = 0.021 and HR = 4.157, CI = 1.915-7.421, *P* = 0.008, respectively). Statistically significant results were also obtained for advanced clinical stage and differentiation status, whereas all other parameters were not significant for overall and progression-free survival (Tables 2 and 3).

**Table 2:** The univariate and multivariate should be listed above the line, respeciteively

	univariateMultivariate			
variables	HR(95%CI)	*p*	HR(95%CI)	*P*
**Tissue miR-21 expression**	4.736(2.035-7.541)	0.016*	3.519(1.411-5.702)	0.021*
**Age at diagnosis**	1.215(0.817-1.571)	0.441	1.194(0.592-2.156)	0.125
**Gender**	2.132(0.451-2.518)	0.585	1.690(0.732-2.215)	0.325
**Clinicalstage at diagnosis**	2.715(1.239-4.215)	<0.001*	2.562(1.148-3.276)	0.009*
**Differentiation**	3.412(1.526-5.164)	0.011*	2.753(1.241-4.517)	0.004*
**Tumor origination**	1.645(0.714-2.211)	0.238	2.142(0.822-2.712)	0.417

CI: confidence interval; HR: hazard ratio;

* Significant relation of clinical factors with overall survival

**Table 3:** Univariate and Multivariate analysis of clinical parameters in relation to progression-free survival

	univariateMultivariate			
**variables**	HR(95%CI)	*p*	HR(95%CI)	*P*
**Tissue miR-21 expression**	3.271(1.518-5.591)	<0.001*	4.157(1.915-7.421)	0.008*
**Age at diagnosis**	1.341(0.719-1.816)	0.318	1.561(0.417-2.167)	0.487
**Gender**	1.714(0.633-3.219)	0.613*	2.154(0.811-3.217)	0.326
**Clinicalstage at diagnosis**	5.154(2.192-10.145)	<0.001*	4.219(2.326-7.619)	<0.001
**Differentiation**	3.272(1.571-4.419)	0.007*	2.145(1.481-3.295)	0.021*
**Tumor origination**	1.826(0.542-2.109)	0.611	1.434(0.553-1.819)	0.525

CI: confidence interval; HR: hazard ratio;

* Significant relation of clinical parameters with progression-free survival

## DISCUSSION

Aberrant miRNAs expression patterns have been described in various cancers and alterations in expression of certain miRNAs correlate highly with progression, diagnosis, and prognosis of specific human malignant diseases. However, the molecular mechanisms by which miRNAs modulate tumor growth are still unknown. In this regard, miR-21 has been identified to function as an oncogene because it is overexpressed in multiple types of malignancy such as colon [37, 38], gastric [39], and lung cancers [40]. More importantly, it has been reported that serum miR-21 may be clinically valuable as a noninvasive diagnostic and prognostic tool in patients with colorectal cancer [34]. In particular, it has been demonstrated that miR-21 expression is elevated in Cholangiocarcinoma cell and tissue samples [27, 41]. However, the diagnostic and prognostic role of miR-21 in ICC, and its underlying mechanisms of action, have not been comprehensively or systematically investigated in previous studies.

In the present study, we identified a diagnostic capacity of serum miR-21 in ICC patients. We also confirmed that miR-21 promoted cell proliferation and tumor growth via direct repression of *PTEN* and *PTPN14* expression. The identification of miR-21 as an important regulator of tumor cell proliferation, apoptosis, cell cycle, and tumor growth in *vitro* and in *vivo* emphasizes an essential role of miR-21 in mediating carcinogenesis of ICC and tumor cell behavior and provides further insight into the contribution and mechanism of altered miR-21 expression in promoting malignant phenotypes. Moreover, our results showed increased miR-21 expression was significantly associated with adverse clinicopathological features. We additionally demonstrated that miR-21 expression levels were also predictive of overall and progression-free survival. Most significantly, our results provided convincing evidence that miR-21 is an independent predictor of poor prognosis in ICC.

MiR-21 has been shown to be overexpressed in multiple types of solid tumors, including cholangiocarcinoma. It is likely that miR-21 plays a fundamental role in malignant transformation and tumor cell behavior. Inhibition of miR-21also has been shown to increase tumor growth *in vivo* [42]. Moreover, miR-21 has been reported to have anti-apoptotic properties in glioblastoma [43] and to modulate Gemcitabine-induced apoptosis in cholangiocarcinoma [29]. Due to the critical functions of its target proteins in various signaling pathways, miR-21 plays an important role in tumor progression and apoptosis by regulating specific target mRNAs, and we identified *PTEN* and *PTPN14* as direct targets of miR-21 in ICC. PTEN and PTPN14 have been shown to play crucial roles in cell proliferation and tumor growth by regulating mTOR and Hippo signaling pathways [44, 45]. These latter proteins are key agents in molecular and cellular pathways regulating organ size through their respective roles in control of cell growth and proliferation [46].

*PTEN* is a ubiquitous tumor suppressor gene and the functional inactivation of PTEN by regulation of its expression is relevant to many solid tumors. Loss of functional PTEN leads to increased activity of AKT and mammalian target of rapamycin (mTOR) kinase pathways, which promote both cell survival and proliferation through phosphorylation and inactivation of multiple downstream mediators [47]. Thus, modulation of expression of PTEN in ICC can impact on the activity of critical downstream mediators of cell proliferation and tumor progression. PTPN14 is a non-receptor tyrosine phosphatase and has been shown to interact with dephosphorylated β-catenin. PTPN14 is also a YAP regulator which can suppress YAP activity by promoting its cytoplasmic localization, and PTPN14 downregulation can phenocopy YAP overexpression in human mammary epithelial cells, further enhancing its transforming phenotype [44]. YAP is a critical component of the mammalian Hippo pathway, which is highly conserved from fly to humans. The Hippo pathway regulates cell numbers by inhibiting cell proliferation and promoting apoptosis through phosphorylation of the transcriptional co-activator YAP in mammals [48]. Additionally, it has been demonstrated that the Hippo-YAP pathway is an upstream regulator of PTEN [45] – which itself is a negative regulator of mTOR - indicating that these two pathways might be coordinately regulated to drive cell growth and cell proliferation.

Our results demonstrated both *PTPN14*, the YAP regulator, and *PTEN* were the direct targets of miR-21. MiR-21 has been shown to play an important role in cell proliferation and cell growth in ICC and other solid tumors from our results and in previous studies. Our observations also indicated that *PTPN14* and *PTEN* overexpression could reverse many of the biological effects of miR-21, which implicates each as primary targets of miR-21 in these processes. In view of our experimental findings, we speculate that the two mRNAs targeted by miR-21 may either perform unrelated functions or may serve to reciprocally regulate each other. In light of our findings and those of previous studies, we propose the hypothesis that miR-21 is an important regulator of mTOR and Hippo pathways, with miR-21 promoting cell proliferation and cell growth. This mechanism might be modulated by YAP regulation of PTPN14, and PTEN, with these two pathways coordinately regulated to enhance the effects of miR-21 on cell behavior and malignant phenotypes. We also demonstrated that patients with PTEN^high^/PTPN14^high^ tumors had significantly longer overall- and progression-free survival than patients whose tumors overexpressed either one or neither of these proteins. These results provide further evidence to support our hypothesis that the two targets interact with each other to magnify the effects of miR-21 on cell behavior and tumor phenotypes.

Although our current results indicate that miR-21 may be a promising screening tool and prognostic biomarker for ICC, we acknowledge that there are three issues requiring further verification. First, miR-21 has been identified in many solid cancers besides ICC, including colorectal cancer, glioblastoma, pancreatic and breast cancers, underscoring the need for vigilance regarding organ and disease specificity while investigating serum miR-21 as a solitary biomarker for ICC. As a consequence, it may be challenging to differentiate whether circulating miR-21 is specifically associated with ICC itself or if this is a common phenomenon that manifests during progression of many cancers as a result of perturbation of the host immune response [49]. In the present study, we addressed this issue by demonstrating that miR-21 levels fell after surgical removal of the ICC in a subset of patients, highlighting the likely validity of miR-21 as a specific biomarker for ICC. Further research surrounding this issue is required to substantiate our initial findings. Second, the functional relationship between PTPN14 and PTEN requires more exhaustive investigation. Drawing from our results and those of previous studies, we conclude that potential crosstalk exists between PTPN14 and PTEN. More specific and targeted experiments to verify and clarify the underlying mechanism between PTPN14 and PTEN are indicated. Resolution of mTOR and Hippo signaling pathway involvement in modulation of miR-21 expression by a negative-feedback mechanism is also required. Third, the potential use of miR-21 expression levels as a diagnostic and prognostic biomarker must be evaluated in diverse ethnic populations because the clinical materials analyzed in this study were solely from patients of Chinese origin (although the fundamental molecular mechanisms we propose are highly unlikely to exhibit significant ethnic variability).

In conclusion, our results provide compelling evidence for the potential utility of miR-21 as an effective noninvasive screening and prognostic tool in patients with ICC. We also provide convincing results demonstrating that miR-21 promotes cell proliferation and growth *in vivo* and *in vitro* by directly targeting *PTPN14* and *PTEN* mRNAs in ICC. Enhanced understanding of process such as tumor cell proliferation and apoptosis that are regulated by miR-21, and the identification of critical targets for individual miRNAs such as *PTPN14* and *PTEN*, provides novel insights into the mechanisms of carcinogenesis and progression in ICC.

## MATERIALS AND METHODS

### Patients and specimens

Serum- and tissue-based specimen collections and studies thereof were approved by the Research Ethics Committee of Xi'an Jiaotong University and Shaanxi Provincial Tumor Hospital. All patients provided written consent and indicated willingness to donate blood and tissue samples for research. A total of 74 patients were enrolled in this study. 57 patients received curative resection and 17 received palliative resection at the First Affiliated Hospital, Xi'an Jiaotong University (Xi'an, China) and Shaanxi Provincial Tumor Hospital between 2007 and 2009. No enrolled patients underwent radio- or chemotherapy before surgery. All tumors were clinically and histologically diagnosed as intrahepatic Cholangiocarcinoma. Inclusion criteria for all cases included: (i) unambiguous histology and absence of mixed tumor types; (ii) absence of any treatment prior to surgery; (iii) age of tissue block less than 7 years. The clinicopathological characteristics of patients are given in Table 1.

### Cell culture

Human ICC cell lines HUCCT1 and RBE were obtained from the Cell Bank of Chinese Academy of Sciences (Shanghai, China), where they were characterized by mycoplasma detection, DNA-fingerprinting, isozyme detection and cell viability measurement. Cells were cultured in RPMI-1640 (Invitrogen, Carlsbad, CA) medium supplemented with 10% fetal bovine serum (Hyclone, Logan, UT) at 37°C in a humidified atmosphere containing 5% CO_2_

### RNA extraction and reverse-transcription quantitative PCR (RT-qPCR)

For miRNA quantification, total miRNA was extracted from cells using miRNeasy RNA isolation Kit (Qiagen, Valencia, CA, USA), according to the manufacturer's instructions. ABI TaqMan miRNA RT-qPCR (Applied Biosystems, Foster City, CA, USA) kits were used to detect and quantify miRNA expression. Data were analyzed with ABI 7500 Fast Real-Time software v.2.0.1 with automatic Ct setting for adapting baseline and threshold for Ct determination. The universal small nuclear RNA U6 (*RNU6B*) was used as an endogenous control for miRNA levels. Each sample was amplified as three replicates and amounts of PCR products produced were normalized to *RNU6B*.

### Oligonucleotide transfection, western blotting, MTT assay, flow cytometric analysis of apoptotic cells and luciferase assays

The miR-21 inhibitors and negative control oligonucleotides were transfected into ICC cells for MTT and apoptosis assays, western blotting, and luciferase assays as previously described [50].

### Colony formation assay

Briefly, 10 cm dishes were seeded with 200 viable cells in complete medium and allowed to grow for 24 h. After transfection with miR-21 inhibitors or negative controls for 48 h, the medium was removed, and cells were washed in PBS and incubated for a further 10 days in complete medium. Each treatment was done in triplicate. The colonies obtained were washed with PBS and fixed in 4% formalin for 10 min at room temperature and then washed with PBS followed by staining with 0.2% crystal violet.

### Soft agar colony formation assay

Cells seeded on a six-well plate were covered with a layer of 0.6% agar in 1640 medium supplemented with 10% fetal bovine serum. After transfection for 48 h, cells were trypsinized, gently mixed with 0.3% agar medium mixture containing selective antibiotics and re-seeded in triplicate into six-well plates. After 4 weeks, the antibiotic-resistant colonies were stained with 0.2% crystal violet and cells counted under microscopy.

### Xenograft model in nude mice

For tumorigenesis assays, we engineered RBE cells to stably express low levels of miR-21, using a lentiviral-based system (pLVTHM). Xenograft tumors were generated by subcutaneous injection of RBE cells (2×10^6^), including RBE-Vector and RBE-miR-21 inhibitors, into the hind limbs of 4-6week old BALB/C athymic nude mice (nu/nu; Animal Center of Xi'an Jiaotong University, Xi'an, China; n = 5 for each group). All mice were housed and maintained under specific pathogen-free conditions, and all experiments were approved by the Animal Care and Use Committee of Xi'an Jiaotong University and performed in accordance with institutional guidelines. Tumor size was measured using a slide caliper and tumor volume was determined by the formula: 0.44 × A × B^2^, where A represents the diameter of the base of the tumor and B represents the corresponding perpendicular value.

### In situ hybridization of miR-21 and scoring

Hybridization procedures in this study used RNA&ISH kits (Roche, USA) with some modifications. In this method, glassware was washed, rinsed with distilled deionized water, and autoclaved before use. Gloves were worn when handling glassware or slides to prevent RNase contamination. Deparaffinized sections mounted on Denhardt's-coated glass slides were treated with Pepsin for 30 min at 37°C. Treated sections were then processed for *in situ* hybridization at 50°C for 24 h. The hybridization mixture contained the labeled oligonucleotide probe, 50% formamide, 10 mmol/L Tris-HCl, 1 mmol/L Vanadyl-ribonucleoside complex (Sigma, USA), 1 mmol/L CTAB (Sigma, USA), pH 7.0, 0.15 mol/L NaCl, 1 mmol/L EDTA, pH 7.0, 1 x Denhardt's mixture and 10% dextran sulfate. Post hybridization, slides were washed three times for 30 min in 0.1 mol/L TBS at 37°C, then treated with 1% blocking reagent (Roche, USA) in TBS with 0.03% Triton X-100 for 30 min at room temperature and incubated for 30 min with an anti-digoxigenin alkaline phosphatase conjugated antibody (Roche, USA) diluted 1:400 in TBS, 0.03% Triton X-100, 1% blocking reagent. After washing three times for 15 min in TBS, 0.05% Tween, slides were rinsed in DAP-buffer and hybridization signals were visualized using nitroblue tetrazolium and 5-bromo-4-chloro-3-indolyl phosphate as substrates in DAP-buffer, 10% PVA (Sigma, USA). Finally, slides were counterstained using nuclear red fast. Staining was scored using a 0-3+ scale. 0 means no staining; 0-1+, trace staining that is weaker than 1+ but stronger than 0. 1+, 2+ and 3+ indicate increased intensity of the staining. We identified 0, 0-1+, and 1+ as negative, and 2+ and 3+ as positive. Sub-regions excluding necrosis, macrophages and infiltrating neutrophils and lymphocytes were selected and scored. The intensity score for an array spot is the average of all its sub-regions.

### Immunohistochemistry and scoring

Paraffin-embedded sections of tumor tissues were stained for PTPN14 and PTEN expression. Immunohistochemistry (IHC) and the scoring system for PTPN14 (1:200, SAB2700311, Sigma-Aldrich, USA) and PTEN (1:100, ab31392, Abcam, UK) were performed as we previously described [50].

### Statistical analyses

Statistical analysis was performed using IBM SPSS statistical software (version 21.0, IBM Corp., Armonk, NY, USA). Mann-Whitney U analyses of variance were used to evaluate statistical differences in serum miRNA expression between unpaired groups. The Wilcoxon test was used to compare miR-21 expression in paired serum samples obtained before surgical tumor resection and 7 days after resection. Receiver operating characteristic (ROC) analysis was performed to determine the diagnostic performance of miR-21expression levels in distinguishing patients with ICC from healthy control subjects. Sensitivity against 100% specificity was plotted for each cutoff threshold, and the area under the curve (AUC) values that reflect the probability of correctly identifying ICC patients from control subjects were computed. The optimal cutoff thresholds for diagnosis were obtained using the Youden index (J). By using the optimal cutoff value, sensitivity, specificity, and positive and negative predictive values were calculated. Survival curves were estimated using the Kaplan-Meier method, and distributions were evaluated by the long-rank test. Cox proportional hazard models of factors related to survival were used to calculate HRs and identify factors affecting survival. Differences in characteristics between the two groups were examined by χ2 and Fisher's exact tests. All *P*-values were determined from 2-sided tests, and statistical significance was based on a *P*-value of 0.05.

## SUPPLEMENTARY MATERIAL, FIGURES



## Abbreviations

ICC: Intrahepatic Cholangiocarcinoma
miRNA: MicroRNAs
3′-UTR: 3′-untranslated region
PDCD4: Programmed cell death 4
PTEN: Phosphatase and tension homolog deleted on chromosome 10
TIMP3: Tissue inhibitor of metalloproteinase 3
RECK: Reversion-inducing cysteine-rich protein with Kazal motifs
PTPN14: Protein tyrosine phosphatase non-receptor type 14
qRT-PCR: Quantitative Real-time Polymerase Chain Reaction
IHC: Immunohistochemistry
ROC: Receiver operating characteristic
AUC: Area under the curve
