# Supplementary material for: MiR-21 promotes intrahepatic cholangiocarcinoma proliferation and growth in vitro and in vivo by targeting PTPN14 and PTEN
Source: Oncotarget. 2015 Feb 28;6(8):5932–46. doi: 10.18632/oncotarget.3465 (PMC4467412; doi:10.18632/oncotarget.3465)
Supplement: Supplementary file 1 [file oncotarget-06-5932-s001.pdf]

MiR-21 promotes intrahepatic cholangiocarcinoma proliferation and growth in vitro and in vivo by targeting PTPN14 and PTEN

Supplementary Material

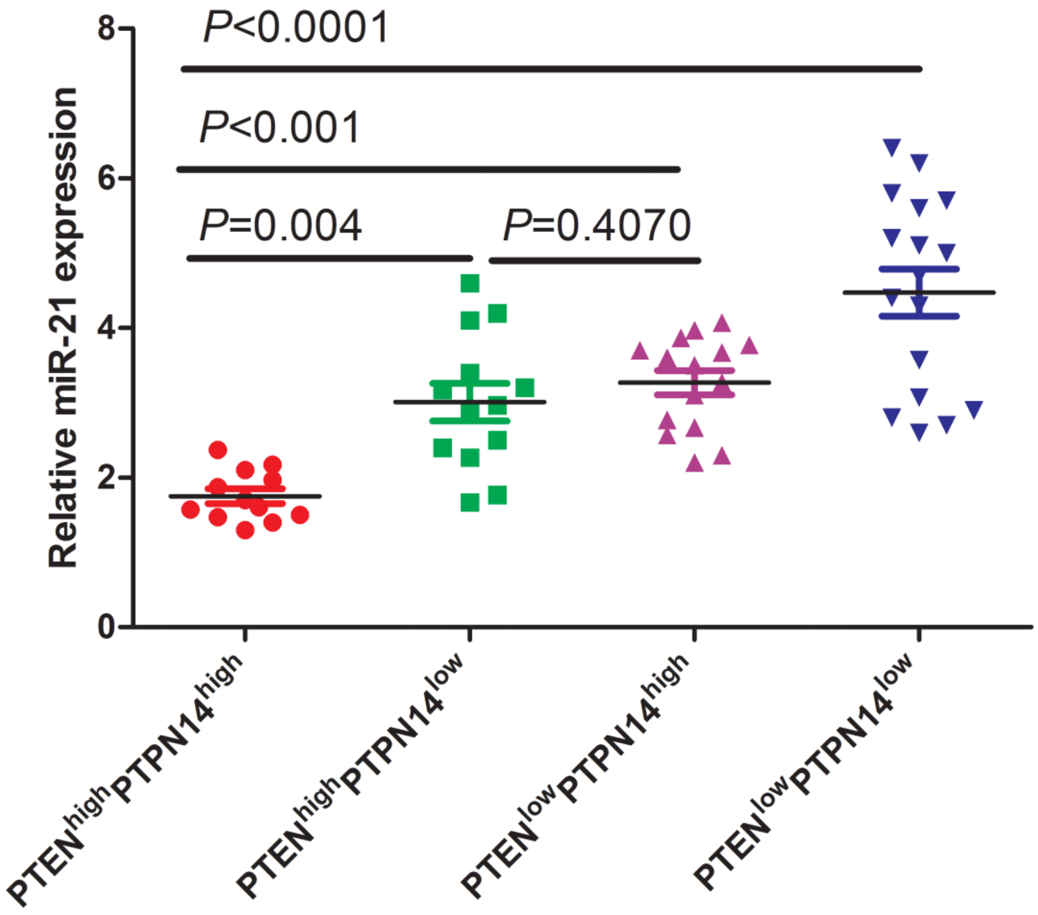

Supplementary Figure 1: Relative miR-21 levels assessed by real-time PCR analysis in ICC tissues stratified according to expression levels of PTPN14 and PTEN.

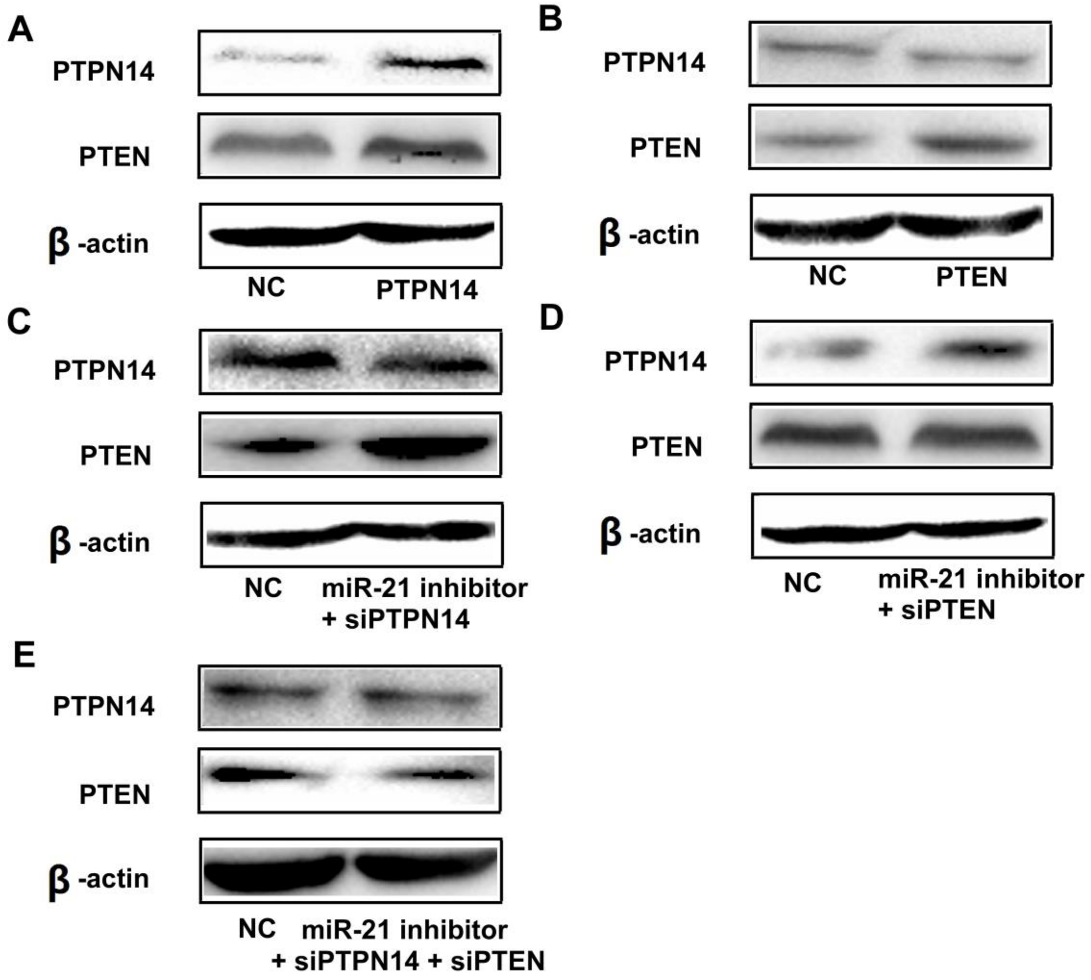

Supplementary Figure 2: Protein levels of PTEN and PTPN14 in RBE cells.

(A) Protein levels of PTEN and PTPN14 in RBE cells transfected with PTPN14 vector. **(B)**Protein levels of PTEN and PTPN14 in RBE cells transfected with PTEN vector. **(C)** Protein levels of PTEN and PTPN14 in RBE cells transfected with miR-21 inhibitor and siPTPN14. **(D)** Protein levels of PTEN and PTPN14 in RBE cells miR-21 inhibitor and siPTEN. **(E)** Protein levels of PTEN and PTPN14 in RBE cells transfected miR-21 inhibitor, siPTEN and siPTPN14.
